# Practice report: an Alberta Métis model for COVID-19 vaccine delivery

**DOI:** 10.17269/s41997-021-00603-7

**Published:** 2022-01-05

**Authors:** Keith D. King, Reagan Bartel, Ashton James, Shannon E. MacDonald

**Affiliations:** 1 University of Alberta, Edmonton, AB Canada; 2 Métis Nation of Alberta, Edmonton, AB Canada

**Keywords:** COVID-19, Vaccination, Indigenous, Métis, Service delivery, COVID-19, vaccination, Autochtones, Métis, prestation de services

## Abstract

**Setting:**

In January 2021, the COVID-19 vaccine became available to First Nations, Métis, and Inuit (FNMI) over the age of 65 living in First Nations communities or Métis settlements in Alberta. In March, vaccine eligibility in Alberta expanded to include FNMI peoples of younger ages and in urban settings. The Métis Nation of Alberta (MNA) and other Indigenous organizations recognized that FNMI populations might be better served by tailored vaccine programs.

**Intervention:**

The MNA is the government for the Métis people in Alberta. During the COVID-19 pandemic, the MNA has supported its citizens, through financial and mental wellness support, access to personal protective equipment, and messaging regarding public health orders. When vaccines became available, culturally appropriate virtual vaccine information sessions were provided. In March 2021, the MNA delivered the first Métis-led COVID-19 vaccination clinic. Unique to the clinic’s success was the location, online booking process, and community presence. The clinic focused on cultural safety, including the availability of Indigenous health professionals to community members, and cultural reference points throughout the clinic.

**Outcomes:**

In the first MNA clinic, over 1300 people were vaccinated. Visitors shared appreciation for the culturally specific aspects of the clinic, which contributed to increased safety and comfort.

**Implications:**

Based on the success of the first Métis-led vaccination clinic, similar services in communities with high numbers of Métis people have been approved. This innovative practice initiative could provide a model of COVID-19 vaccine service delivery that could be used to meet the needs of Métis citizens in other jurisdictions in Canada.

## Résumé

**Lieu:**

En janvier 2021, le vaccin anti-COVID-19 est devenu accessible aux Premières Nations, Inuits et Métis (PNIM) de 65 ans et plus vivant dans les communautés des Premières nations ou les établissements métis de l’Alberta. En mars, l’admissibilité au vaccin en Alberta a été étendue aux personnes des PNIM plus jeunes et vivant en milieu urbain. La Nation métisse de l’Alberta (NMA) et d’autres organismes autochtones ont pensé que les populations PNIM seraient peut-être mieux servies par des programmes de vaccination adaptés.

**Intervention:**

La NMA est le gouvernement des Métis en Alberta. Durant la pandémie, la NMA a appuyé ses citoyens en leur offrant des mesures d’aide financière et de bien-être mental, en leur donnant accès à de l’équipement de protection individuelle et en diffusant des messages sur les ordonnances de santé publique. Quand les vaccins sont devenus disponibles, des séances d’information virtuelles culturellement appropriées ont été offertes. En mars 2021, la NMA a monté la première clinique de vaccination contre la COVID-19 dirigée par des Métis. Le succès de cette clinique a reposé sur son emplacement, sur le processus de prise de rendez-vous et sur la présence communautaire. La clinique a mis l’accent sur la sécurisation culturelle, notamment en mettant à la disposition des résidents des professionnels de la santé autochtones et en plaçant des références culturelles à plusieurs endroits.

**Résultats:**

Au cours de la première clinique de la NMA, plus de 1 300 personnes se sont fait vacciner. Les visiteurs ont dit apprécier les aspects culturellement spécifiques de la clinique, qui ont contribué à en accroître la sécurité et le confort.

**Conséquences:**

Avec le succès de la première clinique de vaccination dirigée par des Métis, des services semblables ont été approuvés dans des communautés où les Métis sont nombreux. Cette initiative novatrice pourrait être un modèle de prestation de services de vaccination contre la COVID-19 adaptés aux besoins des citoyens de la Nation métisse dans d’autres provinces et territoires du Canada.

## Introduction

With the introduction of safe and effective vaccines against COVID-19 in December of 2020 (Government of Canada, 2021a, 2021b), the prioritization of Indigenous people by the National Advisory Committee on Immunization (Government of Canada, 2020) has significantly impacted the federal and provincial governments’ implementation of vaccination programs in Canada. As a result, the particular approach that was taken by provincial and territorial governments, alongside the various First Nations, Inuit, and Métis governments, has varied substantially. This paper describes the unique approach taken through a partnership between the Alberta Ministry of Health, the provincial health service provider (Alberta Health Services or AHS), and the Métis Nation of Alberta (MNA) Health Department in implementing Canada’s first Métis-led COVID-19 immunization clinic.

## Setting

It is estimated that prior to colonization, there were upwards of eight million Indigenous people living in what is now North America, comprising a complex and diverse array of tribal and cultural groups with distinct legal, linguistic, and social traditions (Denevan, 1992). Under the *Canadian Constitution Act, 1982* (Government of Canada, 2021a, 2021b), Indigenous people in Canada include three distinct and diverse groups: First Nations, Inuit, and Métis peoples. Each has unique histories, heritages, cultural practices, spiritual beliefs, languages, and kinship relations (Cooke & McWhirter, 2011). The Métis emerged as post-contact Indigenous people who arose from marriages between settler-voyageurs and adventurers into the North-West and First Nations community members. The Métis identity formed as a distinct nation and is defined not by geographical borders but through kinship relations and histories across the northern plains of Canada and the United States (McDougall & St.Onge, 2017).

In 1928, the MNA became the government for Métis Albertans, with its geographical and legal boundaries being the province of Alberta. The MNA is governed by a Provincial Council composed of a Provincial President and Vice President, and six regional Presidents and Vice Presidents, who have been democratically elected. This Council works toward the mandate of the MNA, promoting and facilitating the advancement of Métis people through self-reliance, self-determination, and self-management (MNA, 2021). The MNA (2021) defines membership in the Nation as “a person who self-identifies as Métis, is distinct from other Aboriginal peoples, is of historic Métis Nation ancestry and who is accepted by the Métis Nation.” Current citizenship in the MNA registry consists of over 48,000 registered Métis in Alberta (MNA, 2021), although census data suggest upwards of 114,000 self-identified Métis in the province (Statistics Canada, 2017).

In Alberta, the COVID-19 vaccine roll-out began in December 2020, with emergency-approved vaccines offered to key populations, focusing on acute care sites with the highest COVID-19 incidence in major cities (Government of Alberta, 2021). Targeted groups included healthcare workers in intensive care units, respiratory therapists, and staff in long-term care facilities and designated supportive living facilities. In January of 2021, a phased roll-out plan was announced, with phase one targeting a variety of high-risk populations, as well as First Nations, Inuit, and Métis over 65 living in First Nations communities or Métis settlements. This phase included the eight Métis Settlements in Alberta.

Phase two of the vaccine roll-out began on March 15, 2021. It included additional sub-groups (full details available at https://www.alberta.ca/COVID19-vaccine.aspx); lower age limits (15 years younger) for First Nations, Métis, or Inuit people; and inclusion of urban Indigenous (off-reserve or settlement) peoples across the province. It is during this phase that the practice innovation reported here took place.

## Contention/Issue

There are multiple complex and contradictory narratives regarding Indigenous peoples’ attitudes towards COVID-19 vaccination in Canada. There has been much debate about perceptions of and responses to Indigenous peoples’ hesitancy to accept the COVID-19 vaccination, depending on the source. The regional, contextual, historical, and political situatedness of various First Nations, Métis, and Inuit governments significantly impact how vaccine acceptance and hesitancy are created and presented through media narratives of their stories. For example, some news outlets have described Indigenous peoples’ vaccine hesitancy in the context of the historical trauma that many Indigenous communities experienced with forced experimentation and attempted assimilation, leading to mistrust in settler health services (Komadina, 2021). Additionally, some communities have requested additional education from trusted professionals, multiple communication channels for information, including radio, and extra attention to combating misinformation on the internet (Zingel, 2021). On the other hand, a conflicting account from the Canadian Press (2021) argues that there is no evidence that historical trauma informs contemporary vaccine hesitancy in Indigenous communities. Preliminary results from a survey study conducted in December 2020 (the “COVImm study,” PI SE MacDonald) show a small but significant reduction in intention to be vaccinated for COVID-19 in Indigenous participants, as compared to a reference population of non-Indigenous participants (MacDonald, 2021).

In an insightful review by Mosby and Swidrovich (2021), they illustrate the historical and contextual impacts of colonization on the diverse Indigenous peoples in Canada and suggest community-specific interventions to address local concerns and foster improved uptake of the vaccine. These include working with local Elders, community leaders, and health professionals who are part of communities to tailor messaging to the unique nations and histories that they are intended to reach, acknowledging that the risks and benefits of vaccination may be understood differently in each community (Mosby and Swidrovich, 2021). These findings reinforce the need for local solutions to vaccine hesitancy in Indigenous communities.

## Intervention/Innovation/Response

**Fig. 1:**
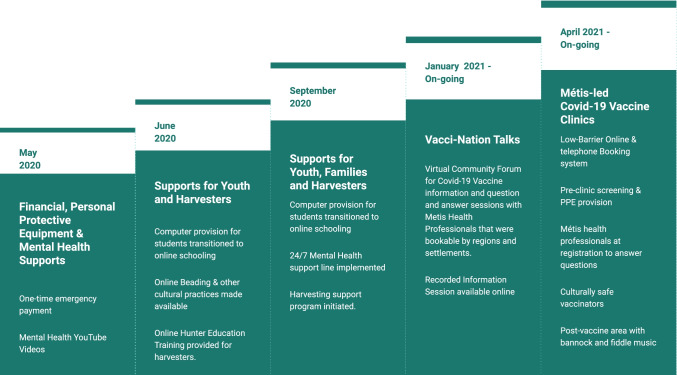
Timeline of Métis Nation of Alberta COVID-19 response

The MNA has been responding to their people’s needs related to COVID-19 since the onset of the pandemic. Early support from the Métis government in Alberta included financial and mental wellness support, access to personal protective equipment including masks and hand sanitizer for their citizens, and ongoing messaging regarding public health orders (http://albertametis.com/covid-19-information/covid-19-resources/). With the introduction of vaccines, regular virtual information sessions with Métis health professionals to answer community questions have been offered to support accurate information sharing and combat misinformation. This offered a culturally safe space to access vaccine-related information (https://www.eventbrite.ca/e/vacci-nation-talks-tickets-144098252753#). Additionally, the MNA website has included up-to-date Frequently Asked Questions (FAQ) documents and a forum for community members to ask questions between information sessions (http://albertametis.com/covid-19-information/covid-19-vaccines-faq/). These and other MNA initiatives are presented in Fig. 1.

In early March 2021, the MNA health department independently applied to the Alberta Ministry of Health’s Public Health and Compliance Division to provide vaccine doses for Métis people living in Alberta. The request was approved, and within 2 weeks, a site in Edmonton was booked by the MNA, and staffing was confirmed by the provincial health authority (Alberta Health Services [AHS]). More than 1300 doses of the COVID-19 vaccine were delivered to eligible Indigenous people over 4 days. Unique to the clinic’s success was the relationship between location (the site of previous Métis celebratory events), the online booking process, the volunteer and MNA staff at pre-registration, registration, pre-vaccination area, provincial health authority vaccinators, and post-vaccination area and exit team. Features specific to this clinic that differentiated it from standard provincial health authority clinics focused on cultural safety, including the availability of Indigenous health professionals to community members, and cultural reference points throughout the clinic, which are described below.

Previous engagement between the MNA health department and MNA citizens indicated varying comfort levels in disclosing their indigeneity to the provincial health authority. In response, the MNA provided a locally controlled online and telephone booking experience focused on respecting Métis data sovereignty and honouring Métis experiences. Appointment information was gathered and retained by the MNA. The MNA visibly branded the booking system to increase trust. A call centre was staffed by the MNA and assisted with booking. At no time was Indigenous status disclosed to AHS or the Alberta Ministry of Health, maintaining data sovereignty through data-sharing agreements.

MNA staff and community were woven throughout the entirety of the clinic. When people seeking vaccination arrived, they were greeted as though at a gathering, making it an experience rather than an appointment. Confirmation of appointment, eligibility, and a COVID-19 screening questionnaire were all completed with the assistance of MNA personnel. The MNA provided masks, and public health measures were monitored by the community rather than AHS staff or security, which differed from other urban clinics. MNA navigators were present throughout to assist Indigenous people as they transitioned from one area of the clinic to another. This included seamless navigation from low-barrier registration to the skilled and welcoming AHS vaccinators. Unique to this clinic was the onsite presence of Métis RNs and Indigenous (and non-Indigenous) physicians. The provision of trusted primary health providers allowed the community members to ask specific vaccine and personal health-related questions. Clinic visitors verbally indicated their appreciation for this innovative addition to the clinic and highlighted that it contributed to their vaccine decision-making.

The provincial health authority staff recognized some of the unique challenges Indigenous people face and provided low-barrier registration and immunization services to non-Métis family members and carers living in the same home. Additionally, high-profile Métis community leaders demonstrated their commitment to the process by being publicly vaccinated and supporting the broader media and social campaign.

Community care continued into the post-vaccination area, which was supervised by MNA staff and a Métis RN. While the purpose of this area was to monitor for syncope and early-onset adverse events, the MNA also provided a slide show highlighting previous community events and Métis fiddle music classics like the *Red River Jig* (Genthon, 2001) playing in the background. These provided visual and auditory cultural cues and entertainment while people waited, which provided familiarity and contributed to attendees consistently staying for their entire 15–30-min post-vaccination waiting period. In addition, sharing food is an important part of Métis gatherings, so clinic participants received a gift of much-loved traditional food (bannock and jam) as they exited the vaccination clinic.

## Outcomes

Over 4 days of this first MNA clinic, 1301 people were vaccinated (see Table 1). Three individuals experienced adverse events, all associated with injection anxiety, which were managed through collaboration with the onsite Métis RNs, the physician, and the AHS vaccination team.

**Table 1:** Self-reported Indigenous status vaccinated at the first MNA clinic

	Number	Percent
Métis	1052	80.9%
First Nations	245	18.8%
Inuit	4	0.3%
Total	1301	100.0%

Creating safe spaces is a journey. This clinic also provided an opportunity to increase AHS vaccinator capacity and provide cultural learning. Daily debriefings allowed the MNA site lead to convey the importance of the clinic and provide learning opportunities to staff working each day. The result was a cadre of AHS staff who verbalized a deeper understanding of the importance of culturally safe vaccine delivery processes. Formal feedback was not obtained; however, social media commentary and MNA videos highlight the overwhelming satisfaction of all those who attended.

## Implications

Diverse media coverage in Canada reflects the complex reasons for COVID-19 vaccine hesitancy. There is no pan-Indigenous response that accurately captures Indigenous peoples’ and nations’ relationships with both colonial and traditional health systems. As such, nation and community-specific interventions should be developed to address these issues (Mosby & Swidrovich, 2021). Given the wide range of vaccine acceptance in the general population in Canada, it is important to consider the additional variability arising from the unique circumstances of Indigenous people. Traditionally, most Indigenous peoples have defined and continue to define well-being far beyond physical health or the absence of disease, emphasizing living a good life or being in balance with their natural relations (Elder Dr. Francis Whiskeyjack, personal communication, November 9, 2020).

Research from LaFrance and Nichols (2008) looked at Indigenous evaluation methods and found four fundamental values emerged to guide this work: being a people of a place, recognizing our gifts, honouring family and community, and respecting sovereignty. Utilizing this framework for evaluating the first Métis-led COVID-19 vaccination clinic, the MNA team recognized the unique situatedness of Métis people in Alberta, offering the clinic in a known location of previous Métis celebrations, in the traditional homeland and close to the MNA government headquarters. For recognizing our gifts, the clinic built on traditional kinship relationships, utilizing volunteers and community members to organize and deliver the services and engage participants through traditional food, music, and support. For honouring family and community, the local health authority recognized some of the unique challenges Indigenous people face and provided low-barrier registration and immunization services to non-Métis family members and carers living in the same home. Additionally, high-profile Métis community leaders demonstrated their commitment to the process by being publicly vaccinated and supporting the broader media and social campaign. Finally, in respecting sovereignty, the service was wholly led, organized, and delivered by the MNA, with immunizer resource support from the provincial health authority. Additionally, data sovereignty was maintained through data-sharing agreements with the Alberta Ministry of Health.

There is growing recognition of the need to create culturally safe spaces that reflect the diversity between and within Indigenous peoples of Canada. The success of this clinic highlights the importance of respectful collaborations between Indigenous nations, provincial and territorial governments, and health authorities. It emphasizes that all public health practitioners have a role in creating culturally safe spaces. Based on the success of the MNA’s first Métis-led vaccination clinic, similar services are being offered in additional communities with high numbers of Métis people across Alberta. To date, 3215 people have been vaccinated through MNA-led COVID-19 vaccine clinics in various settings. This service delivery model is proposed as a potentially useful approach to meeting the COVID-19 vaccination needs of Métis citizens in other jurisdictions in Canada. It is also a call for federal, provincial, and territorial health authorities and governments to reflect on their role in inviting and supporting Indigenous nations and governments to create safe spaces for future vaccine delivery programs.

## Implications for policy and practice

What are the innovations in this policy or program?

- This paper outlines the development of a new practice model for delivering vaccinations to Métis people in Canada with preliminary evidence of success. The response from the community was overwhelmingly positive and suggests that Métis-led clinics may provide improved outcomes for vaccination in Métis communities if enabled to deliver culturally appropriate services.

What are the burning research questions for this innovation?

- To scale up this innovation and evaluate it thoroughly, there needs to be consistent and continued investment in capacity building and collaboration between the Métis Nation of Alberta (and other Métis governments across Canada), the provincial governments, and health service providers. Through this investment, robust evaluation and the development of consistent and substantial support for data collection, data-sharing (that honours data sovereignty), and resources to set up and provide vaccination services on an ongoing basis can be achieved and adequately evaluated.

## Data Availability

The activities and reflections presented in this commentary do not include shareable data.
